# Role of Vitamin E in the Treatment of Alzheimer’s Disease: Evidence from Animal Models

**DOI:** 10.3390/ijms18122504

**Published:** 2017-11-23

**Authors:** Agnese Gugliandolo, Placido Bramanti, Emanuela Mazzon

**Affiliations:** IRCCS Centro Neurolesi “Bonino-Pulejo”, Via Provinciale Palermo, Contrada Casazza, 98124 Messina, Italy; agnesegugli@hotmail.it (A.G.); bramanti.dino@gmail.com (P.B.)

**Keywords:** Alzheimer’s disease, vitamin E, tocopherol, tocotrienol

## Abstract

Alzheimer’s disease (AD) is a neurodegenerative disorder representing the major cause of dementia. It is characterized by memory loss, and cognitive and behavioral decline. In particular, the hallmarks of the pathology are amyloid-β (Aβ) plaques and neurofibrillary tangles (NFTs), formed by aggregated hyperphosphorylated tau protein. Oxidative stress plays a main role in AD, and it is involved in initiation and progression of AD. It is well known that Aβ induced oxidative stress, promoting reactive oxygen species (ROS) production and consequently lipid peroxidation, protein oxidation, tau hyperphosphorylation, results in toxic effects on synapses and neurons. In turn, oxidative stress can increase Aβ production. For these reasons, the administration of an antioxidant therapy in AD patients was suggested. The term vitamin E includes different fat-soluble compounds, divided into tocopherols and tocotrienols, that possess antioxidant action. α-Tocopherol is the most studied, but some studies suggested that tocotrienols may have different health promoting capacities. In this review, we focused our attention on the effects of vitamin E supplementation in AD animal models and AD patients or older population. Experimental models showed that vitamin E supplementation, by decreasing oxidative stress, may be a good strategy to improve cognitive and memory deficits. Furthermore, the combination of vitamin E with other antioxidant or anti-inflammatory compounds may increase its efficacy. However, even if some trials have evidenced some benefits, the effects of vitamin E in AD patients are still under debate.

## 1. Introduction

Alzheimer’s disease (AD) is a fast, progressive neurodegenerative disorder and represents the major cause of dementia in the elderly. The clinical features are memory loss, and cognitive and behavioral impairment [1]. The hallmarks of the pathology are the presence of senile plaques, formed by amyloid-β (Aβ), and neurofibrillary tangles (NFTs), formed by aggregated hyperphosphorylated tau protein [2,3]. The prevalence of AD is 10–30% in the population older than 65 years with an incidence of 1–3%. Familial AD is present in a small percentage of patients that inherited mutations in genes involved in Aβ processing and is characterized by an early onset (about 45 years). However, in the majority of cases (>95% of patients), AD is presented as a sporadic form with a late onset at about 80–90 years, and is caused by defects in Aβ clearance from the brain [4]. In particular, mutations in the genes encoding for presenilin 1 (PSEN1), presenilin 2 (PSEN2) and amyloid precursor protein (APP) are associated with early-onset forms of AD, while ε4 and ε2 variants of the apolipoprotein E *APOE* gene can influence AD susceptibility [5].

Aβ is formed by the cleavage of the APP, a type I membrane glycoprotein. However, APP is not simply the Aβ precursor, but it participates in normal brain functioning. Indeed, even if its role is not completely clear, it is involved in brain development, memory and synaptic plasticity. Three isoforms of APP exist, namely APP_695_, APP_751_ and APP_770_, which are generated by alternative splicing of exons 7 and 8. In the brain, the APP_695_ isoform is mainly expressed [6]. APP can be metabolized by two different pathways: the amyloidogenic and non-amyloidogenic pathways [6]. In the non-amyloidogenic pathway, APP is cleaved by α-secretase at position 17 producing C-terminal fragment (CTF) α and APPsα, that seemed to exert a neuroprotective action. In this case, α-secretase cleaves APP inside the Aβ peptide region, preventing the formation of Aβ [7,8]. Instead, the amyloidogenic pathway involves the consecutive actions of two enzymes, β- and γ-secretases, leading to the production of Aβ. β-secretase cleaved APP producing APPsβ and the CTFβ, C99 [2,7]. The β-secretase 1 (BACE1) is the most important β-secretase in the brain. PSEN1 and PSEN2 play a role in regulating the activity of γ-secretase, and, for this reason, mutations in these genes can increase Aβ formation changing γ-secretase activity, and, as we said above, are associated with early-onset forms of AD. CTFβ is after cleaved by γ-secretase, and at the end Aβ peptides of different sizes were released [2,7]. Aβ_1–40_ is the most abundant form of Aβ in the brain, but Aβ_1–42_ is the more toxic due to its tendency to aggregate and form oligomers, and it is the Aβ isoform associated with AD [9,10,11]. After nucleus formation, the aggregation continues forming oligomers and insoluble fibrils. The fibrils associate, forming plaques, that contain Aβ_1–40_, Aβ_1–42_ and other components [12]. At first, the plaques were considered as the main pathologic element of AD, but now it is thought that the real damaging agent may be represented by Aβ_1–42_ oligomers, whose levels correlate with the severity of the cognitive decline in AD [13,14]. Furthermore, it has been shown that oligomeric Aβ_1–42_ caused a marked increase in oxidative stress markers compared to fibrillar Aβ_1–42_, and, as a consequence of oxidative stress, also increased cell death [15].

The alteration of Aβ brain level in AD may be caused by a variation in its production, but also of its clearance. The clearance is mediated by different pathways, including the activation of degrading enzymes and receptor-mediated cellular and vascular clearance. Among the receptor, low-density lipoprotein receptor related protein 1 (LRP1) mediated clearance of Aβ. Also, P-glycoprotein is involved in Aβ clearance. Aβ is degraded by some peptidases, including neprilysin, insulin-degrading enzyme (IDE) and endothelin-converting enzyme [2].

Oxidative stress represents one of the main factors involved in AD initiation and progression [16]. Indeed, the brain is particularly sensitive to oxidative damage as a consequence of its request of energy and its consumption of oxygen. In particular, in vitro and in vivo studies indicated that Aβ seemed to be the responsible of the increased oxidative stress, increasing the levels of hydrogen peroxide (H_2_O_2_), lipid peroxides, protein oxidation and oxidative stress markers [7,16,17]. In particular, Aβ_1–42_ oligomers can insert into the lipid membrane forming α-helices and start reactive oxygen species (ROS) production, causing lipid peroxidation and protein oxidation [7]. Moreover, it caused a perturbation of the fluidity of the plasma membrane [18]. ROS produced can induce oxidative damage to brain molecules. Different studies showed that cells exposed to Aβ showed an increase in ROS production, inducing cell death [18,19]. De Felice et al. [20] found that Aβ oligomers caused ROS formation through a mechanism involving *N*-methyl-d-aspartate receptor (NMDA-R) activation [20].

In turn, oxidative stress can enhance Aβ production. Indeed, mitochondrial dysfunction, enhancing ROS production, induced high levels of Aβ [21]. Moreover, oxidative stress can enhance Aβ aggregation and facilitate tau phosphorylation and polymerization, promoting the initiation and progression of AD [16].

In addition, it seems that Aβ induced acceleration of tau pathology and about this it has been observed that Aβ_1–42_ oligomers promote tau hyperphosphorylation, resulting in toxic effects on synapses [22,23]. The tau protein acts to stabilize the microtubules. In AD, tau protein is hyperphosphorylated. Hyperphosphorylated tau can decrease its binding to microtubules, and induced tau aggregation, forming NFTs. Hyperphosphorylated tau interferes with neuronal functions, and caused microtubule destabilization and damaged axonal transport [24].

## 2. Vitamin E

The term vitamin E refers to different fat-soluble compounds present in plants, that can be divided in tocopherols and tocotrienols, that may be differentiated for the presence of the unsaturated side-chain, presenting three double bonds. Each class has four homologs and the eight members are distinguished as α-, β-, γ-, and δ-tocopherol, and as α-, β-, γ-, and δ-tocotrienol based on the number and localization of methyl groups [25].

Vitamin E is an essential micronutrient for humans, useful for maintaining the integrity of cell membranes. The daily dietary intake recommended for vitamin E vary between 3 and 15 mg in different countries and varies depending on age [26]. Seeds and edible oils, such those by almond, peanuts, olive, palm oil, canola, corn, and soybean, contain high levels of tocopherols and tocotrienols, while plant foods containing low lipid levels, such as fruit and vegetables, have a scarce quantity [27].

The main vitamin E role is its antioxidant action, given that it is the most important lipophilic radical scavenging antioxidant in vivo. Vitamin E scavenges free radicals mainly by hydrogen atom transfer reaction obtaining a non-radical product and a vitamin E radical, that may react with another radical to give a stable product, attack lipids, or react with a reducing agent such as vitamin C or ubiquinol to regenerate vitamin E [28]. α-tocopherol is the most studied because it is the main form of vitamin E in tissues. α-tocopherol acts as chain-breaking antioxidant in lipoproteins and cell membranes, limiting lipid peroxidation and maintaining membrane integrity [26]. However, some studies revealed that tocotrienols have different health improving properties and may have a better antioxidant capacity compared to α-tocopherol [29,30].

Vitamin E is an important factor for other processes and for tissue and organ development, including brain. In addition to the potent antioxidant action, it was observed that vitamin E can act as signaling and gene regulation molecule, and vitamin E incorporation into cell membranes can influence the activity of membrane-associated and integrated proteins modulating their signaling via molecular mechanisms that are independent from the antioxidant activity [26].

Vitamin E has a pivotal role in brain. Indeed, the pathological manifestations of the familial syndrome ataxia with vitamin E deficiency (AVED), caused by mutations in the α-tocopherol transfer protein (TTP) gene *TTPA* in humans, and in the *Ttpa^−^/^−^* knock-out mouse model, are represented by neurological symptoms [31]. TTP regulates levels and distribution of α-tocopherol and a high expression of TTP was found in the brain, indicating that this protein supply antioxidant protection to the brain, given its high vulnerability [32]. Moreover, vitamin E half-life in the brain is slow compared to other organs, suggesting the existence of specific mechanisms acting to regulate central nervous system (CNS) vitamin E levels and the concentrations of α-tocopherol vary in different brain areas [32].

The problems associated with vitamin E deficits may be caused by the loss of its antioxidant protection, and consequently ROS accumulation that compromised the integrity and function of biological membranes. At cellular level, vitamin E deficiency caused degeneration of neurons. In particular to study the importance of α-tocopherol in the cerebellum, *Ttpa^−^/^−^* mice were used, in which the TTP protein is lacking and represent a model of vitamin E deficiency and oxidative stress. Even if these mice received a diet supplemented with α-tocopherol, only plasma levels of vitamin E appeared normal, while its levels in the cerebellum and prefrontal cortex showed only a slight increase, suggesting a main role of TTP in the brain. Vitamin E deficiency induced an increase in cerebellar oxidative stress, as suggested by the high levels of protein nitrosylation, prevented in part by dietary vitamin E supplementation. Moreover, vitamin E deficiency caused cellular atrophy and diminished dendritic branching of Purkinje neurons associated with cognitive deficits that were prevented by vitamin E supplementation. All together, these data suggested that an adequate vitamin E level is essential for anatomic integrity and physiological function of the Purkinje neurons [33]. Furthermore, vitamin E deficits are associated not only to cell damage, but also with impaired motor coordination, cognitive functions, ataxia and lipid peroxidation [33,34,35]. In particular, lipid peroxidation in *Ttpa^−^/^−^* mouse brains showed a significant increase, while α-tocopherol supplementation prevented lipid peroxidation and neurological symptom development [34]. Fukui et al. [36] demonstrated that the long term vitamin E deficiency caused cognitive deficits in mice through the increase in brain oxidation. Mice were fed with a vitamin E deficient-diet from one month to three or six months of age. In the vitamin E-deficient three-month-old mice, a decline in cognitive function was observed. In addition, in six-month-old vitamin E-deficient mice, lipid peroxidation products in the cerebral cortex, cerebellum and hippocampus significantly increased. Serum cholesterol content significantly increased in both vitamin E-deficient groups of mice [36].

## 3. Vitamin E and Alzheimer’s Disease (AD)

Interestingly, studies in vitro showed that vitamin E may be able to counteract oxidative stress induced by Aβ [37,38]. In particular, Yatin et al. [38] demonstrated that vitamin E was able to prevent Aβ_1–42_ induced protein oxidation, ROS production, and neurotoxicity in primary rat embryonic hippocampal neuronal culture, maybe through the scavenging of Aβ-induced free radicals [38]. Furthermore, Tamagno et al. [39] showed that Aβ peptides induced oxidative stress, as demonstrated by the increase in 4-hydroxynonenal (HNE), H_2_O_2_, lipid peroxidation and thiobarbituric acid reactive substances (TBARS) production, and was accompanied by apoptosis, increasing caspase 3 activation, cytochrome c release and cleavage of poly-ADP ribose polymerase (PARP). Interestingly, these effects were prevented by α-tocopherol [39]. In addition to decreased cell viability and oxidative stress, exposition of cells to Aβ_1–42_ caused a significant reduction in phospholipid and ubiquinone-10 levels, as well as in α3 and α7 subunit levels of nicotinic acetylcholine receptors (nAChRs). However, vitamin E pretreatment partially prevented these alterations. These results indicated that Aβ induced lipid peroxidation may cause the alterations in membrane lipid composition and the decreased expression of nAChRs involved in the pathogenesis of AD [40].

Moreover, oxidative stress induced Aβ-like substances and apoptosis in rat hippocampus, resulting in cognitive deficit. However, memory deficits and apoptosis were prevented by vitamin E supplementation, thanks to its antioxidant action [41].

Aβ_1–42_ reduced the surface expression of the main glutamate transporter in adult brain, glutamate transporter-1 (GLT-1), in mouse astrocytes through oxidative stress, and caused GLT-1 ubiquitination and mislocalization at the cell membranes, provoking a prolonged extracellular lifetime of released glutamate, but this effect was prevented by the water-soluble analog of vitamin E trolox [42].

Aβ-induced oxidative damage caused neurotoxicity also against neural progenitor cells (NPCs). Indeed, Aβ_1–40_ blocked the development and differentiation of NPCs, associated with elevated levels of protein carbonyls and lipid peroxidation products, such as HNE and malondialdehyde (MDA) and accumulated Aβ monomers and oligomers. Treatment with vitamin E partially abolished these effects, suggesting that Aβ influences neurogenesis through oxidative damage, but vitamin E was not able to counteract the reduced formation of neuronal processes [43].

However, some of the biological effects of vitamin E did not depend on its antioxidant action, but it influenced the expression of genes involved in AD. Indeed, it was found that vitamin E deficiency influenced gene expression in the hippocampus and in particular, the genes regulated by vitamin E were associated with hormones and hormone metabolism, nerve growth factor (NGF), apoptosis, dopaminergic neurotransmission, clearance of Aβ and advance glycated end products [44]. In particular, in this study, rats received a vitamin E deficient or standard diet for 9 months. Rats receiving the diet without vitamin E showed a reduced expression of NGF, a neurotrophin that promotes neuron survival. Among the genes involved in dopaminergic system, the sodium-dependent dopamine transporter and D3 dopamine receptor were down-regulated in rats fed with a diet without vitamin E. Furthermore, these rats showed a decreased expression of APP binding protein member 1, that binds and stabilizes APP, and of preproalbumin, a precursor of albumin that binds and transports Aβ [44].

### 3.1. Effects of Vitamin E Supplementation in AD Animal Models

The important role of oxidative stress in AD was already demonstrated, but it is important to notice that oxidative stress occurs early in AD, even before Aβ plaque formation. Indeed, using different AD models, it was demonstrated the increase of oxidative stress, as indicated by increased lipid peroxidation [45,46]. Moreover, also an increase in the activity of antioxidant enzymes, namely glutathione peroxidase and superoxide dismutase (SOD) was observed [46]. Notably, these alterations preceded the formation of Aβ plaques and NFTs, indicating the early development of oxidative stress in AD [45,46]. Interestingly, in association with the increased levels of oxidative stress markers, a reduction of vitamin E levels, together with reduced glutathione (GSH) were observed [46]. These results, other than showing the importance of oxidative stress as a pathogenic mechanism in AD, suggested that the use of antioxidant compounds may be useful for AD prevention/treatment. In particular, vitamin E, whose reduction was demonstrated in the aforementioned AD model, thanks to its potent antioxidant action, may be a suitable compound able to prevent or delay disease progression. With the aim of evaluating the efficacy of vitamin E supplementation, different experimental studies were carried out that demonstrated that vitamin E exerts health benefits.

The action of vitamin E was tested against Aβ toxicity in rats infused with Aβ_1–42_, showing that its oral administration, from three days before Aβ infusion, prevented learning and memory deficits [47]. However, in this study Aβ treated rats did not show an increased oxidative stress and the antioxidant action of vitamin E was not demonstrated.

Aβ_1–42_-treated mice showed memory deficits and increased oxidative stress, demonstrated by the increased activities of the cytosolic Cu, Zn-SOD and mitochondrial Mn-SOD in the hippocampus and cerebral cortex in association with increases in MDA, a marker of lipid peroxidation, and protein carbonyl in the same areas. However, α-tocopherol, administrated orally, from seven days before Aβ injection, significantly attenuated Aβ_1–42_ induced effects, including the memory deficits [48].

Nishida et al. [49] studied the effects of α-tocopherol deficiency in a double-mutant mouse model obtained crossing *Ttpa^−^/^−^TTP* knockout mice, where the lack of α-tocopherol in the brain caused oxidative stress, with *APPsw* AD transgenic mice. The double-mutant *Ttpa^−^/^−^APPsw* mice receiving a α-tocopherol-deficient diet developed a more severe and precocious cognitive dysfunction in the Morris water maze, novel-object recognition and contextual fear conditioning tests compared to *APPsw* mice, together with an increase in Aβ deposits. However, α-tocopherol supplementation to *Ttpa^−^/^−^APPsw* mice partially prevented the development of cognitive dysfunction and decreased Aβ deposits in cortex and hippocampus. However, even if an improvement of the condition was obtained, the recovery was not complete, maybe because only 10% of α-tocopherol can be recovered in the brain of *Ttpa^−^/^−^* mice even after α-tocopherol supplementation compared with wild-type (WT) mice [49]. Moreover, the same group demonstrated that oxidative stress caused by α-tocopherol deficiency damaged Aβ clearance from the brain and blood, causing as a consequence Aβ accumulation in *Ttpa^−^/^−^APPsw* mouse brain and plasma, that was reduced by α-tocopherol supplemented diet [50]. However, α-tocopherol deficiency did not influence Aβ generation, given that *APP* mRNA and protein levels of C-terminal fragments of APP-β, -α, and -γ/ε in the brain of *Ttpa^−^/^−^APPsw* mice were not increased compared with *APPsw* mice. Instead, Aβ accumulation was due to a decreased Aβ clearance. Indeed, the expression level of the Aβ-degrading peptidase IDE was decreased in *Ttpa^−^/^−^* mouse brain. In addition, the Aβ aggregation increased in the brain of the *Ttpa^−^/^−^* mice compared with WT. *Ttpa^−^/^−^APPsw* mice showed a marked increase of both plasma Aβ_1–40_ and Aβ_1–42_ levels, that was partially reduced in mice receiving the α-tocopherol-supplemented diet. The Aβ accumulation in the plasma was also caused by impaired clearance from the blood. Indeed, decreased levels of LRP-1, the Aβ receptor that transports Aβ into the hepatocytes, were found in the plasma membrane fraction of *Ttpa^−^/^−^* mouse liver, causing a reduced Aβ clearance in the blood, and this may explain the increased Aβ levels in *Ttpa^−^/^−^APPsw* mouse plasma [50]. All together, these results demonstrated that vitamin E deficiency in AD models caused cognitive deficits and Aβ accumulation, but vitamin E supplementation reverted these conditions, at least partially.

Plasma phospholipid transfer protein (PLTP) plays a main role in vitamin E transfer and its deficiency reduced brain vitamin E levels in mice. *PLTP*-knockout (*PLTP*-KO) mice showed an increase in oxidative stress and brain Aβ_1–42_ levels, while the expression of the synaptic function marker synaptophysin was reduced. Moreover, *PLTP*-KO mice presented memory deficits a week after Aβ_25–35_ injection. Dietary supplementation of vitamin E in *PLTP*-KO mice prevented Aβ_25–35_-induced memory deficits and oxidative stress [51].

Sung et al. [52] pointed the attention on the importance of the timing of vitamin E administration. Indeed, an early vitamin E administration reduced Aβ_1–40_ and Aβ_1–42_ levels and amyloid deposits in younger Tg2576 mice that received a diet supplemented with vitamin E from five months of age, but not in older AD mice, receiving the same diet from 14 months of age, when amyloid plaques are already deposited. However, vitamin E supplementation could counteract oxidative stress induced in both groups, reducing 8,12-*iso*-iPF2α-VI levels in cortex and hippocampus as a result of the increased vitamin E brain level [52].

Other than on Aβ deposits, vitamin E exerted a positive action on tau hyperphosphorylation, the other hallmark of AD. Indeed, transgenic mice overexpressing human tau protein developed filamentous tau aggregates in the CNS, but supplementation with α-tocopherol delayed the development of tau pathology, decreased oxidative stress and improved motor function [53]. The protein tau become hyperphosphorylated by the action of different kinases, including p38 [54]. In an in vitro study, primary cortical neurons incubated with Aβ showed p38 activation, leading to tau hyperphosphorylation [55]. Moreover, post-mortem brains of AD patients showed that phospho-p38 mitogen-activated protein kinase (MAPK) immune reactivity was present at early stage of the disease [56]. Giraldo et al. [55] demonstrated in vitro that Aβ-induced activation of p38 MAPK that leads to tau hyperphosphorylation, but these effects were prevented when neurons were co-incubated with trolox [55]. Moreover, in vivo, APP/PS1 transgenic mice exhibited higher levels of phospho-p38 in the hippocampus compared to WT animals, but not in the cortex. This increase was abolished when the animals received a vitamin E-supplemented diet [55].

These studies indicated that vitamin E may be able to counteract Aβ-induced effects and tau hyperphosporilation, acting in this way on both AD hallmarks. But, more important from a clinical point of view is vitamin E action on cognitive performance.

Variants of the *APOE* gene are associated with an increased risk of developing AD. Apolipoprotein E (Apo E)-deficient mice, receiving a diet supplemented with α-tocopherol for 12 months, showed a better behavioral performance compared to those receiving a normal diet. The better performance was associated with the preservation of the dendritic structure in vitamin E-treated Apo E-deficient mice. Moreover, these mice showed normal levels of lipid peroxidation and glutathione. These results indicated that the dietary supplementation with vitamin E exerted a protective action against oxidative insults in Apo E-deficient mice and prevents the deficits in cognition and neuropathologic alterations [57]. This study associated the prevention of cognitive deficits to the antioxidant action of vitamin E. In line with these results, Ishihara et al. [58] found that α-tocopherol improved cognitive function, decreasing oxidative stress. Transgenic AD mice showed decreased levels of α-tocopherol and GSH while oxidized glutathione (GSSG) and lipid peroxidation were increased in the cerebral cortex and hippocampus at 4 months of age. However, the dietary supplementation with α-tocopherol mitigated the reduction of GSH levels and the increase of GSSG and TBARS. Furthermore, mice showed cognitive impairment at six months of age, but α-tocopherol supplementation could improve cognitive function. Brain redox state was evaluated by magnetic resonance imaging using 3-hydroxymethyl-proxyl as a probe, and it showed an increase in reactive radical production in the brains of AD mice at 4 months of age, that was attenuated by α-tocopherol supplementation. These findings indicated that oxidative stress can be associated with the cognitive impairment and α-tocopherol may improve cognitive function, decreasing oxidative stress [58].

Moreover, the chronic treatment for 15 days with trolox showed a trend toward a reduction of oxidative stress induced from Aβ plaque and toward a reversal of the structural changes in dystrophic neurites associated with Aβ plaque [59]. However, in this case the reduction was not significant, and it may indicate the need of longer treatments or higher doses of vitamin E.

RRR-α-tocopherol quinone, an oxidative metabolite of α-tocopherol, was reported to attenuate Aβ induced citotoxicity, inflammation and oxidative stress in vitro, blocking Aβ_42_ fibril formation [60]. In vivo, α-tocopherol quinine (α-TQ) treatment could reduce Aβ oligomers levels, but not total Aβ and Aβ fibrillar oligomers in AD mouse brains. In addition, microglial activation was inhibited by α-TQ, via the block of NF-κB pathway and, consequently, the levels of the pro-inflammatory cytokines interleukin 6 (IL-6) and interleukin 1β (IL-1β) were decreased. Moreover, α-TQ administration counteracted oxidative stress, increasing SOD activity, decreasing the levels of inducible nitric oxide synthase (iNOS) and MDA, a marker of lipid peroxidation in comparison with AD mice. Moreover, α-TQ administration improved memory and cognitive dysfunction [61].

Repetitive concussive brain injury (RCBI) is known to enhance brain Aβ accumulation and behavioral dysfunction, in parallel with brain lipid peroxidation [62]. Conte et al. [63] tested the effect of vitamin E supplementation in Tg2576 mice, a mouse model of AD-like brain amyloidosis overexpressessing mutant human APP, subjected to RCBI. In particular, from 11 months of age, mice were divided into groups to receive a normal chow or chow-supplemented with vitamin E for four weeks, and after were subjected to RCBI. The same diets were maintained for another eight weeks post-injury. Animals receiving vitamin E showed an increase in vitamin E brain levels while brain lipid peroxidation levels decreased. After RBCI, mice receiving vitamin E supplemented diet did not show an increase in Aβ peptides while learning deficits were mitigated in comparison with animals fed with regular chow. These results suggested that the increased brain oxidative stress following RCBI accelerated Aβ accumulation and behavioral deficits in Tg2576 mice, but vitamin E, suppressing lipid peroxidation, prevented Aβ accumulation and behavioral impairments in these mice [63].

Even if α-tocopherol is the most investigated compound of the vitamin E family, more recently also tocotrienols were investigated for their protective effects, sometimes showing a more prominent action than that exerted by the most famous α-tocopherol. α-tocopherol and tocotrienol were tested against streptozotocin (STZ). In particular, intracerebroventricular injection of streptozotocin in subdiabetogenic dose in rats induced cognitive impairment and oxidative and nitrosative stress, but rats that received oral administration of α-tocopherol and tocotrienol presented an improvement of the cognitive function. Moreover, α-tocopherol and tocotrienol administration prevented the reduction of GSH and SOD and catalase activity and reduced MDA, nitrite and cholinesterase activity in the brains of STZ treated rats in a dose dependent manner. Notably, all these results showed a more potent action of tocotrienol [64].

The tocotrienol-rich fraction (TRF), containing different vitamin E analogs obtained from palm oil was tested on APPswe/PS1dE9 double transgenic mice. The TRF was shown to be able to block Aβ fibrils and Aβ oligomers formation in vitro in a dose dependent manner. In addition, the daily TRF supplementation for 10 months mitigated Aβ depositions in the cortex and thioflavin-*S*-positive fibrillar type plaques in the hippocampus and cortex of APPswe/PS1dE9 double transgenic mice and improved cognitive function. However, TRF supplementation did not exert anti-inflammatory action, showing no action on the microglia activation and the authors suggested that this may be caused by insufficient dose, suboptimal method of administration, low bioavailability or late age of initiation (five months) [65].

A summary of the experimental models evaluating vitamin E supplementation is shown in Table 1.

### 3.2. The Combined Treatment with Vitamin E and Other Compounds in AD Animal Models

In some studies, vitamin E treatment was associated with other molecules with antioxidant or anti-inflammatory effects. In this way, the different compounds can act synergistically to exert a stronger protective effect.

Tg2576 mice, a mouse model of AD, received a diet supplemented with α-tocopherol and indomethacin, an anti-inflammatory drug, for seven months: from 8 (before Aβ deposition) to 15 months of age (when Aβ deposits are abundant). The animals receiving the supplemented diet showed increased brain levels of vitamin E and a suppression of brain oxidative stress and inflammation, as demonstrated by the reduction of glial fibrillary acidic protein (GFAP), IL-1β, cortex and hippocampus levels of prostaglandin E_2_ (PGE_2_), thromboxane A_2_ (TxB_2_), and of isoprostane F_2α_-VI (iPF_2α_-VI), and protein carbonyls, as biomarkers of lipid peroxidation and protein oxidation, respectively. The anti-inflammatory and antioxidant effects were associated with the decrease of soluble and insoluble Aβ_1–40_ and Aβ_1–42_ and Aβ deposits in neocortex and hippocampus. These results showed that the two drugs had an additive effect on brain inflammation and oxidative stress [66].

Given that there is a synergistic relationship between vitamins C and E, because vitamin C recycles vitamin E radicals regenerating its antioxidant properties [67], the effect of the combination of different doses of vitamin E with vitamin C was tested in APP/PSEN1 mice. It was observed that the combination of a high dose of vitamin E with vitamin C increased vitamin E levels in liver and brain, but was not able to improve cognitive function and was less effective in reducing oxidative stress. With the aim to verify if vitamin E supplement was too high, and negatively influenced oxidative stress and cognitive function, other experiments were carried out with a lower vitamin E dose. The low vitamin E dose with vitamin C treatment improved spatial memory deficits in APP/PSEN1 mice and mice receiving the supplements had lower neuroprostane levels than control animals. However, amyloid deposition was not influenced by vitamin treatment [68]. These data indicated that oxidative stress and memory can be improved by vitamin supplements even if aggregated amyloid proteins in the brain were not influenced.

The combination therapy with folic acid and α-tocopherol was evaluated in mice injected in the lateral ventricle with Aβ_1–40_. The treatment with 25 mg/kg of folic acid and 250 mg/kg of α-tocopherol, or 50 mg/kg of folic acid and 500 mg/kg of α-tocopherol, induced a significant improvement of spatial learning deficits induced by Aβ_1–40_, while higher doses did not exert protection against the Aβ_1–40_-induced cognitive impairment. The combination of folic acid with α-tocopherol exerted a protective action against the Aβ_1–40_-induced cognitive decline through a reduction of synaptic dysfunction. Indeed, while Aβ_1–40_-induced synaptic loss in mice, as demonstrated by the reduction of synaptophysin levels, the treatment with folic acid and α-tocopherol prevented the reduction in synaptophysin levels in the hippocampus. Moreover, the injection of Aβ_1–40_ caused neuroinflammation with the activation of astrocytes and microglial cells, as indicated by the increased number of GFAP and CD68-positive cells, respectively. However, the treatment did not have effects on neuroinflammation, given that it did not influence astrocyte activation and the number of CD68-positive cells in the hippocampus. Interestingly, the combination of folic acid and α-tocopherol exerted antioxidant effects, as demonstrated by the capacity of inhibiting the Aβ_1–40_-induced iNOS and neuronal nitric oxide synthase (nNOS) upregulation and the prevention of the Aβ-induced neuronal death in the mouse hippocampi. Moreover, Aβ_1–40_ injected mice showed an increased activity of the mitochondrial complexes I, II, and IV, that was decreased by the treatment with folic acid (50 mg/kg) and α-tocopherol (500 mg/kg), except for complex II activity [69].

The administration of a combination of *N*-acetylcysteine, α-lipoic acid and α-tocopherol to aged rats from 18 months to 22–24 months of age prevented oxidative stress, inflammation and age-dependent changes in synaptosomal parameters together with a reduction of lipid peroxidation and it improved learning and memory functions [70,71]. The same combination was tested in aged rats to evaluate its action on Aβ. In particular, Sinha et al. [72] demonstrated an increase in the expression and protein levels of APP in the brain cortex of 22–24 months old rats, together with a higher activity of β-secretase and a reduction of neprilysin activity, an enzyme involved in the degradation of amyloid protein, in comparison with 4–6 months old rats. Consequently, older rats presented an accumulation of Aβ_42_ in brain cortex. However, rats that received from the age of 18 months a dietary supplementation with a mix of *N*-acetylcysteine, α-lipoic acid and α-tocopherol showed a reduction of APP, β-secretase activity and Aβ_42_ compared to rats with a normal diet, while neprilysin activity increased. Moreover, the supplemented diet prevented the spatial learning and memory impairments [72].

A summary of the experimental models evaluating vitamin E supplementation together with other compounds is shown in Table 2.

### 3.3 Vitamin E Supplementation in AD Patients

Even if experimental studies indicated that vitamin E exerts beneficial effects in AD animal models, its efficacy in AD patients is still debated. Indeed, even if AD patients showed lower plasma and cerebrospinal fluid levels of vitamin E [73,74], while higher plasma levels were associated with a reduced risk of AD [75], the results of studies on AD patients are not clear. Indeed, only some studies evidenced benefits from vitamin E supplementation (Table 3). Sano et al. [76] showed in a cohort of patients affected by moderately severe AD, that vitamin E (2000 IU/day) delayed AD progression. In particular, a delay in the time to the primary outcome (death, institutionalization, loss of the ability to perform basic activities of daily living, severe dementia) was reported for the patients receiving α-tocopherol compared to the placebo group. However, no improvements in cognitive function were observed, but it may be caused by the severity of AD in this cohort. Accordingly, another study demonstrated in patients with mild to moderate AD that vitamin E (2000 IU/day) reduced functional decline and caregiver burden. No significant adverse effects were reported in the α-tocopherol group [77].

In contrast with these data, other reports showed contrasting results, showing not convincing evidence that vitamin E treatment was able to exert positive effects in AD [78]. A three-year study showed that vitamin E administration did not exert beneficial actions in patients with mild cognitive impairment (MCI). In particular, the rate of progression from MCI to AD was evaluated, but vitamin E treatment did not influence the probability of progression to AD [79]. Lloret et al. [80] treated AD patients with 800 IU/day for 6 months. After the treatment, they divided patients in two groups: “respondents”, patients whose GSSG levels decreased after vitamin E administration and maintained the scores in cognitive tests, and “non-respondents”, in which oxidative stress did not decrease after vitamin E treatment. Moreover, in non-respondent patients, vitamin E administration may be detrimental given that the cognitive function of these patients decreased more than in patients treated with placebo. The authors speculated that in this case vitamin E could act as a pro-oxidant [80]. A systematic review concluded that no evidence showed that vitamin E improved cognitive function or prevented the progression of dementia, but it might slow functional decline in AD and, notably, vitamin E did not increase the risk of developing serious side effects or mortality [81].

Kryscio et al. [82] evaluated whether vitamin E (400 IU/day) could prevent dementia in asymptomatic older men obtaining a negative result. However, a reason may be the low incidence of AD and dementia found during follow-up [82]. However, the vitamin E dose administrated was lower than in other studies.

One of the main concern about vitamin E treatment was that high doses may increase mortality. Indeed, a meta-analysis indicated that high doses of vitamin E (≥400 IU/day) might increase the risk of mortality [83]. However, Dysken et al. [77] did not find an increase in the mortality rate in AD patients receiving vitamin E compared to those receiving placebo, on the contrary it was reduced. Accordingly, the study of Pavlik et al. [84] in a cohort of patients taking high doses of vitamin E reported that vitamin E did not increase mortality. Indeed, patients receiving a treatment including vitamin E (2000 IU/day) survived longer compared to those treated with cholinesterase inhibitors or with no drugs [84]. Moreover, a meta-analysis showed no relationship between dose and risk of mortality and in particular, vitamin E did not have effects on all-cause mortality at doses up to 5500 IU/day [85].

Other studies evaluated whether vitamin E from food may be able to exert beneficial effects against AD. A higher consumption of foods with an elevated content of vitamin E could modestly decrease the risk of dementia and AD [86] and was associated with a reduced cognitive decline in older persons [87]. Morris et al. [88] examined if vitamin E from food or individual tocopherols could exert a protective action against AD and cognitive decline. The results showed that higher intakes of vitamin E from food were associated with a reduced incidence of AD and a slower decline in cognitive scores. Among the different form of tocopherols, α- and γ-tocopherols were associated with a slower cognitive decline and reduced AD risk. In particular, the results suggested that the combined intake of the different tocopherol forms, may be more useful than α-tocopherol alone in the protection against AD. Then, the lack of association between AD and vitamin E administration reported in some studies might be because, normally, supplements contained only α-tocopherol. Interestingly, the association between the brain concentrations of tocopherols and Aβ was investigated. γ-tocopherol levels were associated with reduced Aβ load. Moreover, it was found that high α-tocopherol levels were associated with elevated Aβ load when γ-tocopherol concentration was reduced, and with reduced Aβ load when γ-tocopherol concentration was high [89]. A similar result suggesting that the protective action of vitamin E may be exerted by a mix of the different vitamin E forms instead of α-tocopherol alone was obtained by Mangialasche et al. [75], that showed that high plasma levels of vitamin E were associated with a lower risk of AD in advanced age. In particular, they found in a cohort of subjects older than 80 years, that those having plasma levels of total tocopherols, total tocotrienols or total vitamin E in the highest tertile presented a lower risk of developing AD compared to subjects in the lowest tertile.

All these results may indicate that a combination of tocopherols and not an individual tocopherol may have a stronger neuroprotective action against AD development. For this reason, given that other tocopherols except α-tocopherol are less studied other studies are needed.

Another trial did not report advantages in cognitive function in women receiving vitamin E compared to those receiving placebo. However, it is interesting to notice that vitamin E group showed less cognitive decline compared with the placebo group that included women with a low dietary intake of vitamin E (lower than 6.1 mg/day), but not compared with women receiving placebo with a high intake of dietary vitamin E [90].

Interestingly, the combination of vitamin E with other antioxidants, such as vitamin C, was shown to be able to exert beneficial actions in AD. In particular, high intakes of vitamins E and C from food or from supplements were associated with a lower risk of cognitive decline and AD [91,92,93]. A study showed that older women receiving long-term supplements of vitamin E and vitamin C had a better cognitive function compared to women who had never used vitamin E or C supplements and the scores were higher increasing the duration of the treatment. Instead, the treatment with vitamin E alone was associated with modest cognitive benefits [94]. However, another study found that vitamin E and/or vitamin C supplement did not delay the development of dementia or AD. However, a limitation of this study was the lack of information about doses and duration of the treatment [95].

Given that previous studies did not show consistent evidence of a positive effect of vitamin E supplementation, the British Association for Psychopharmacology stated that vitamin E can not be recommended for AD treatment or prevention [96].

Several factors may influence and explain the different and conflicting results obtained by different studies. First, vitamin E dose is an important variable that needs to be evaluated. The successful results were obtained using high doses of vitamin E. Then, in some reports, vitamin E dose used was not high enough to obtain a clinical result. However, it is not easy to determine the optimal dose and further studies are needed, but it is important to notice than high doses do not seem to be associated with increased mortality and side effects. A second important point is the vitamin E form used, given that in most cases supplements contained α-tocopherol, but it seems that other dietary tocopherols and tocotrienol, and then their combination, may be more useful to obtain a stronger neuroprotection. Another point that should be addressed is the timing of study initiation. Indeed, it is possible to speculate that an early administration may be more useful in preventing cognitive deficits, given that no study demonstrated the ability to reverse the disease process. In this context, the severity of AD is also an important factor to consider and that may influence the success of the therapy. In addition, long-term treatment seems to be necessary to obtain a significant result.

Moreover, it must be considered that not all the studies considered the same parameters to evaluate the success of the vitamin E treatment, and for this reason the results of different studies cannot be compared to each other. Indeed, while some studies evaluated the capacity to carry out daily life activities, others considered the progression of MCI to AD. In addition, confounding variables cannot be excluded, such the use of other drugs or the presence of concomitant diseases, that may influence the results.

Notably, an interesting hypothesis was proposed by Cervantes and Ulatowski [97] in their review that single nucleotide polymorphisms may have a role in responsiveness to vitamin E treatment. They suggest that clinical trials should be designed considering a “personalized medicine approach” to predict individual’s responsiveness to vitamin E.

## 4. Conclusions

The number of people affected by AD is continuously increasing and, for this reason, a treatment able to prevent or delay the progression of the disease is needed. Evidence showed that oxidative stress plays a main role in AD pathology. In particular, it was observed that Aβ takes part in increasing oxidative stress, causing ROS production and leading to lipid peroxidation and protein oxidation. In turn, oxidative stress promotes Aβ production. Given this, antioxidant compounds may be helpful in the prevention/treatment of AD. Vitamin E is one of the most important antioxidant and some data indicated that it could counteract Aβ-induced oxidative stress. Evidence from preclinical studies showed that vitamin E administration may be beneficial in AD. Indeed, vitamin E is not only able to reduce Aβ-induced oxidative stress, but also able to improve memory and cognitive deficits. In particular, the precocious and prolonged administration seemed to be related to better results. Even if α-tocopherol is the most investigated member of vitamin E family, also tocotrienols showed good results in AD animal models, exerting in some cases a stronger action than α-tocopherol. Furthermore, the combination of vitamin E with other compounds with anti-inflammatory or antioxidant activity, that act synergistically, may be useful for the treatment of AD. However, clinical trials provided conflicting results about vitamin E efficacy in the prevention and/or treatment of AD. High vitamin E doses and prolonged supplementation seem to be associated with better results. Moreover, a higher intake of foods rich in vitamin E, which contain a combination of different forms of vitamin E, was associated with a better cognitive function. However, further studies are required to obtain clear results about vitamin E efficacy in AD. In this context, trials evaluating different doses and forms of vitamin E and their combination could be helpful.

## Figures and Tables

**Table 1:** Experimental studies evaluating vitamin E supplementation.

Animal Model	Vitamin E Administration	Results	Ref.
Wistar rats infused with Aβ_1–42_ (300 pmol/day)	α-tocopherol (150 mg/kg) was administered orally for 23 consecutive days, from 3 days before the start of Aβ_1–42_ infusion	Vitamin E prevented learning and memory deficits.	[47]
C57BL/6 mice with intracerebroventricular injection of Aβ_1–42_	α-tocopherol (150 mg/kg) was administered orally in a volume of 1 mL/kg for 27 days, starting 7 days before Aβ_1–42_ injection	α-tocopherol significantly attenuated Aβ_1–42_ induced oxidative stress and memory deficits	[48]
*Ttpa^−^/^−^APPsw* mice	α-tocopherol supplemented diet (750 mg/kg)	α-tocopherol partially reversed the onset and severity of cognitive dysfunction and decreased Aβ deposits	[49]
*Ttpa^−^/^−^APPsw* mice	α-tocopherol supplemented diet (750 mg/kg)	α-tocopherol deficiency impaired Aβ clearance from the brain and blood, causing Aβ accumulation in *Ttpa*^−^*/*^−^*APPsw* mouse brain and plasma, that was reduced in mice fed with α-tocopherol supplemented diet	[50]
*PLTP*-KO mice with intracerebroventricular injection of oligomeric Aβ_25–35_ peptide	Vitamin E-supplemented chow diet (800 mg/kg α-tocopherol acetate)	Vitamin E supplemented diet prevented Aβ_25–35_-induced memory deficits and oxidative stress in *PLTP*-KO mice	[51]
Tg2576 mice	Vitamin E supplemented diet (2 IU/g; diet average intake of vitamin E was ~8–10 IU/day) from 5 to 13 or 14 to 20 months of age	Early vitamin E administration reduced Aβ levels and amyloid deposits. Vitamin E supplementation reduced 8,12-*iso*-iPF2α-VI level	[52]
Tau transgenic mice	α-tocopherol-supplemented diet (160 or 1500 IU/kg)	α-tocopherol improved tau pathology and motor function, and decreased oxidative stress	[53]
APP/PS1 transgenic mice	α-tocopheryl acetate supplemented diet (800 IU/kg) for 21 days	Vitamin E abolished the increase in phospho-p38 (MAPK) levels in the hippocampus	[55]
Apolipoprotein E-deficient mice	1% of α-tocopherol added to the mouse chow for 12 months	Better behavioral performance associated with reduced oxidative stress. Preservation of the dendritic structure	[57]
Transgenic mice expressing the mutant human genes *APP*, *presenilin1* and *tau*	Mice were divided in two groups and received a diet supplemented with α-tocopherol (1.342 mg/g diet) or a normal diet (0.076 mg α-tocopherol/g diet) from 2 to 6 months of age.	Dietary supplementation with α-tocopherol mitigated the reduction of GSH levels and the increase of GSSG and TBARS. Moreover, α-tocopherol supplementation improves cognitive function in 6 months old AD mice. α-tocopherol supplementation decreased the levels of reactive radicals in the brains.	[58]
APPswe/PS1d9 mice	Trolox (210 mg/kg) administered by gavage for 15 days	Trolox showed a trend toward a reduction of Aβ plaque-induced oxidative stress and of structural changes in neurites	[59]
APPswe/PS1dE9 mice	α-tocopherol quinine (100 mg/kg) administered by gavage daily for 4 weeks	α-tocopherol quinine reduced Aβ oligomers levels in AD mouse brains. Microglial activation was inhibited by α-TQ, blocking NF-κB pathway. α-TQ administration counteracted oxidative stress and improved memory and cognitive dysfunction	[61]
Tg2576 mice subjected to RCBI	Mice received a regular chow or chow-supplemented with vitamin E (2 IU/g diet) for 4 weeks, and subjected to RCBI. The same diet was maintained for 8 weeks post-injury	Mice receiving vitamin E supplemented diet showed increased vitamin E brain levels and decreased brain lipid peroxidation levels. After RBCI, mice receiving vitamin E did not show an increase in Aβ peptides while learning deficits were mitigated	[63]
Intracerebroventricular streptozotocin (STZ) (3 mg/kg) treated rats	Oral administration of α-tocopherol (100 mg/kg) and a mixture of α-, β-, γ-tocotrienol (50 and 100 mg/kg) for 21 days starting from the day of STZ injection	α-tocopherol and tocotrienol improved cognitive impairment, prevented the reduction of GSH and catalase, reduced MDA, nitrite and cholinesterase activity in the brains of STZ rats in a dose dependent manner. Tocotrienol showed a stronger action	[64]
APPswe/PS1dE9 mice	TRF contained α-tocotrienol (196.0 mg/g), β-tocotrienol (24.0 mg/g), γ-tocotrienol (255.0 mg/g), *δ*-tocotrienol (75.0 mg/g), and α-tocopherol (168.0 mg/g) and was orally administered to mice (60 mg/kg body weight) daily from 5 to 15 months of age	TRF blocks Aβ fibrils and Aβ oligomers formation in vitro in a dose dependent manner. In addition, TRF mitigated Aβ depositions, thioflavin-*S*-positive fibrillar type plaques and improved cognitive function, but TRF did not exert anti-inflammatory action	[65]

AD: Alzheimer’s disease; Aβ: Amyloid-β; α-TQ: α-Tocopherol quinine; GSH : Reduced glutathione; GSSG: Oxidized glutathione; MAPK: Mitogen-activated protein kinase; MDA: Malondialdehyde; PLTP-KO: Phospholipid transfer protein-knockout; RCBI: Repetitive concussive brain injury; STZ: Streptozotocin; TBARS: Thiobarbituric acid reactive substances; TRF: Tocotrienol-rich fraction.

**Table 2:** Experimental studies evaluating vitamin E supplementation together with other compounds.

Animal Model	Compound Administration	Results	Ref.
Tg2576 mice	Diet supplemented with α-tocopherol (2 IU/mg diet) and indomethacin (10 mg/L in drinking water) from 8 to 15 months of age. Given that each mouse eats about 4–5 mg chow/day, and drinks 3 to 4 mL water/day, the estimated average vitamin E and indomethacin intake for each animal was ~8–10 IU/day and 30–40 ng, respectively	Mice receiving the supplemented diet presented increased brain levels of vitamin E and a suppression of brain oxidative stress and inflammatory responses (reduction of GFAP, IL-1β, PGE_2_, TxB_2_, iPF_2α_-VI and protein carbonyls). Reduction of soluble and insoluble Aβ_1–40_ and Aβ_1–42_ and Aβ deposits	[66]
APPswe/PS1dE9 mice	Diet supplemented with vitamin C alone (3 g/kg diet) or in combination with a high (750 IU/kg diet) or low (400 IU/kg diet) dose of vitamin E. Considering the normal food intake of the mice, with the high dose of vitamin E diet administration, mice received about 50 mg/kg body weight/day vitamin E, and 100 mg/kg vitamin C.	Vitamin C with the low dose of vitamin E reduced oxidative stress and improved spatial memory deficits. The combination of vitamin C with a high dose of vitamin E was less effective. However, amyloid deposition was not influenced by vitamin treatment.	[68]
Mice subjected to intracerebroventricular injection with Aβ_1–40_	Oral administration of folic acid (25, 50 or 100 mg/kg) with α-tocopherol (250, 500 or 1000 mg/kg), daily for 14 days	The treatment with of folic acid and α-tocopherol improved Aβ_1-40_ induced spatial learning deficits and cognitive decline through a reduction of the synaptic dysfunction process. The combination of folic acid and α-tocopherol exerted antioxidant effects and induced a decrease in the activity of mitochondrial complexes I and IV, but not complex II	[69]
Young (4–6 months) and aged (22–24 months) rats	Diet supplemented with a combination of *N*-acetylcysteine (50 mg/100 g body weight), α-lipoic acid (3 mg/100 g body weight) and α-tocopherol (1.5 mg/100 g body weight) from 18 months until they were 22–24 months old	The combination of *N*-acetylcysteine, α-lipoic acid and α-tocopherol to aged rats prevented oxidative stress and inflammation in the brain	[71]
Young (4–6 months) and aged (22–24 months) rats	Combination of *N*-acetylcysteine (50 mg/100 g body weight), α-lipoic acid (3 mg/100 g body weight) and α-tocopherol (1.5 mg/100 g body weight) administered orally with the diet from 18 months until they were 22–24 months old	The combination of *N*-acetylcysteine, α-lipoic acid and α-tocopherol to aged rats prevented changes in synaptosomal parameters together with a reduction of lipid peroxidation, and improved learning and memory functions	[70]
Young (4–6 months) and aged (22–24 months) rats	Diet supplemented with *N*-acetylcysteine (50 mg/100 g body weight), α-lipoic acid (3 mg/100 g body weight) and α-tocopherol (1.5 mg/100 g body weight) from the age of 18 months until they were 22–24 months old	The supplemented diet with *N*-acetylcysteine, α-lipoic acid and α-tocopherol prevented the spatial learning and memory impairment and these rats showed a reduction of APP, β-secretase activity and Aβ_42_ compared to rats with a normal diet, while neprilysin increased.	[72]

Aβ: Amyloid-β; APP: Amyloid precursor protein; GFAP: Glial fibrillary acidic protein; IL-1β: Interleukin 1β; iPF_2α_-VI: Isoprostane F_2α_-VI; PGE_2_: Prostaglandin E_2_; TxB_2_: Thromboxane A_2_.

**Table 3:** Clinical trials evaluating vitamin E supplementation in AD patients.

Subjects	Vitamin E Treatment	Duration	Results	Ref.
341 AD patients	2000 IU/day *dl*-α-tocopherol	2 years	Vitamin E slowed disease progression.	[76]
613 patients with mild to moderate AD	2000 IU/day of α-tocopherol (*dl*-α-tocopheryl acetate)	6 months to 4 years	Patients treated with α-tocopherol showed a slower cognitive functional decline. No side effects associated with vitamin E.	[77]
769 patients with mild cognitive impairment	1000 IU/day for 6 weeks and after 2000 IU/day	3 years	Vitamin E treatment did not influence AD progression.	[79]
33 AD patients	800 IU/day	6 months	Vitamin E respondents had a lower oxidative stress and did not show loss of cognition. Non-respondents showed a reduction of cognitive function.	[80]

## Abbreviations

AD	Alzheimer’s disease
Aβ	Amyloid-β
NFTs	Neurofibrillary tangles
PSEN1	Presenilin 1
PSEN2	Presenilin 2
APP	Amyloid precursor protein
Apo E	Apolipoprotein E
CTF	C-terminal fragment
BACE1	β-secretase 1
LRP1	Low-density lipoprotein receptor related protein 1
IDE	Insulin-degrading enzyme
H_2_O_2_	Hydrogen peroxide
ROS	Reactive oxygen species
NMDA-R	*N*-methyl-d-aspartate receptor
AVED	Ataxia with vitamin E deficiency
TTP	α-Tocopherol transfer protein
CNS	Central nervous system
HNE	4-Hydroxynonenal
TBARS	Thiobarbituric acid reactive substances
PARP	poly-ADP ribose polymerase
nAChRs	Nicotinic acetylcholine receptors
GLT-1	Glutamate transporter-1
NPCs	Neural progenitor cells
MDA	Malondialdehyde
NGF	Nerve growth factor
SOD	Superoxide dismutase
GSH	Reduced glutathione
WT	Wild-type
PLTP	Phospholipid transfer protein
PLTP-KO	Phospholipid transfer protein-knockout
MAPK	mitogen-activated protein kinase
GSSG	Oxidized glutathione
α-TQ	α-Tocopherol quinine
IL-6	Interleukin 6
IL-1β	Interleukin 1β
iNOS	Inducible nitric oxide synthase
RCBI	Repetitive concussive brain injury
STZ	Streptozotocin
TRF	Tocotrienol-rich fraction
GFAP	Glial fibrillary acidic protein
PGE_2_	Prostaglandin E_2_
TxB_2_	Thromboxane A_2_
iPF_2α_-VI	Isoprostane F_2α_-VI
nNOS	Neuronal nitric oxide synthase
MCI	Mild cognitive impairment
